# A rare presentation of extramedullary multiple myeloma as a large gluteal soft tissue mass: A case report

**DOI:** 10.1016/j.ijscr.2025.111829

**Published:** 2025-08-16

**Authors:** Faten Limaiem, Mohamed Amine Gharbi, Ramzi Bouzidi

**Affiliations:** a University of Tunis El Manar, Faculty of Medicine of Tunis, Tunisia; b Pathology Department, Hospital Mongi Slim, La Marsa, Tunisia; c Department of Orthopedic Surgery, Hospital Mongi Slim, La Marsa, Tunisia

**Keywords:** Extramedullary multiple myeloma, Soft tissue plasmacytoma, CD138, Pathology, Buttock mass, Case report

## Abstract

**Introduction:**

Extramedullary multiple myeloma is a rare condition, occurring in 7 % to 18 % of cases, where malignant plasma cells spread beyond the bone marrow to various organs and soft tissues. This report presents a case of secondary extramedullary multiple myeloma with an unusual soft tissue mass, highlighting diagnostic challenges, management considerations, and the importance of early detection.

**Case presentation:**

A 78-year-old woman with a history of multiple myeloma and AL amyloidosis presented with a rapidly enlarging, non-tender gluteal mass over the past two months. Physical examination disclosed a firm, non-tender mass palpable in the left buttock, measuring approximately 23 cm. Imaging revealed osteolytic lesions in the left hip and a large mass invading surrounding tissues. A surgical biopsy was performed. Histological examination revealed a highly cellular malignant proliferation composed of atypical plasma cells arranged in sheets, surrounded by a thick fibrous capsule. Immunohistochemistry showed strong CD138 positivity, confirming the diagnosis of extramedullary plasmacytoma. The patient was scheduled for adjuvant radiotherapy but passed away due to severe pneumonia shortly after the first session of treatment.

**Clinical discussion:**

The case underscores the diagnostic challenges and importance of a comprehensive evaluation in extramedullary multiple myeloma. Early detection and appropriate management are crucial for optimal patient care.

**Conclusion:**

This case highlights the significance of recognizing and managing extramedullary multiple myeloma, particularly in patients with uncommon soft tissue presentations. Comprehensive evaluation and timely intervention are essential for enhancing patient outcomes in such cases.

## Highlights

• Soft tissue involvement in multiple myeloma is rare, arising from skeletal extension or hematogenous spread, often as large vascularized nodules.
• Extramedullary Multiple Myeloma can mimic other tumors, requiring thorough histopathological and immunohistochemical analysis.
• Despite treatment advances, extramedullary disease remains difficult to manage with limited response to standard therapies.
• Early detection and thorough evaluation are key to improving patient outcomes in Extramedullary Multiple Myeloma.

## 1 Introduction

Multiple myeloma (MM) is a clonal plasma cell malignancy characterized by the uncontrolled proliferation of abnormal plasma cells within the bone marrow, often resulting in end-organ damage such as hypercalcemia, renal impairment, anemia, and bone lesions. According to the World Health Organization and the International Myeloma Working Group, the diagnosis of MM requires ≥10 % clonal bone marrow plasma cells or a biopsy-proven plasmacytoma, in addition to evidence of myeloma-defining events. These include one or more CRAB features (hyperCalcemia, Renal failure, Anemia, Bone lesions) or biomarkers of malignancy such as a clonal bone marrow plasma cell percentage ≥ 60 %, an involved/uninvolved serum free light chain ratio ≥ 100, or more than one focal lesion on MRI [[1], [2], [3]]. Extramedullary multiple myeloma (EMM) is a rare and aggressive variant of MM in which malignant plasma cells infiltrate tissues outside the bone marrow, including soft tissues, lymph nodes, and visceral organs. It may occur at initial diagnosis (primary EMM) or during relapse (secondary EMM), with a reported incidence ranging from 7 % to 18 % [4,5]. Despite improvements in imaging and therapeutic strategies, EMM remains a clinical challenge due to its heterogeneous presentations, poor response to conventional treatments, and unfavorable prognosis, with median survival often less than 12 months [[2], [3], [4], [5]]. Although EMM is increasingly recognized, data on its occurrence in atypical anatomical sites remain sparse. In particular, involvement of soft tissue regions such as the buttock is exceedingly rare and often misdiagnosed, contributing to delayed treatment. This report describes a rare case of secondary extramedullary multiple myeloma presenting as a rapidly enlarging buttock mass, an exceptionally uncommon site of involvement. It aims to underscore the diagnostic challenges and clinical implications of atypical soft tissue presentations in EMM, emphasizing the importance of early recognition to guide timely and appropriate management.

This case report adheres to the SCARE Criteria [6].

## 2 Case presentation

### 2.1 Patient history and presenting complaint

A 78-year-old woman with a known history of MM and AL amyloidosis, previously treated with a bortezomib, lenalidomide, and dexamethasone (VRD) regimen in 2022 achieving complete response, presented with a rapidly enlarging, non-tender gluteal mass that had developed over the past two months. She had been under regular follow-up with the hematology department and outpatient clinic and was not receiving maintenance therapy at the time of recurrence. Her medical history also included well-controlled hypertension. She reported no drug allergies, did not smoke, consume alcohol, or use illicit substances, and lived independently with no notable occupational exposures (Table I).

**Table I:** Timeline of clinical events for patient case.

Date	Event
January 7th, 2022	Treatment of MM and AL amyloidosis with VRD regimen, achieving remission
January 2024	Development of a rapidly enlarging gluteal mass
March 7th, 2024	Presentation to the hospital with complaints of the mass
March 10th, 2024	Imaging (X-ray and MRI) performed
March 11th, 2024	Surgical biopsy conducted
March 15th, 2024	Histopathological diagnosis of extramedullary myeloma confirmed
April 15th, 2024	Initiation of adjuvant radiotherapy
April 25th, 2024	Development of severe pneumonia
April 27th, 2024	Death due to pneumonia

### 2.2 Physical examination findings

The physical examination revealed that the patient was in a deteriorated general condition. Vital signs were stable, with blood pressure, heart rate, respiratory rate, and temperature all within normal limits. The skin showed no signs of jaundice or cyanosis, remaining intact and free of rashes. Notably, a firm, non-tender mass was found in the left buttock, measuring approximately 23 cm; this mass was mobile, well-defined, and exhibited no signs of erythema or warmth. Additionally, there was no lymphadenopathy in the inguinal or pelvic regions. Neurologically, the patient demonstrated no sensory or motor deficits in the lower extremities, and reflexes were normal and symmetrical. The abdominal examination indicated a soft, non-tender, and non-distended abdomen, with no palpable masses or organomegaly. Overall, examinations of the cardiovascular, respiratory, and gastrointestinal systems were unremarkable, suggesting a need for further diagnostic evaluation.

### 2.3 Diagnostic workup

**Fig. 1:**
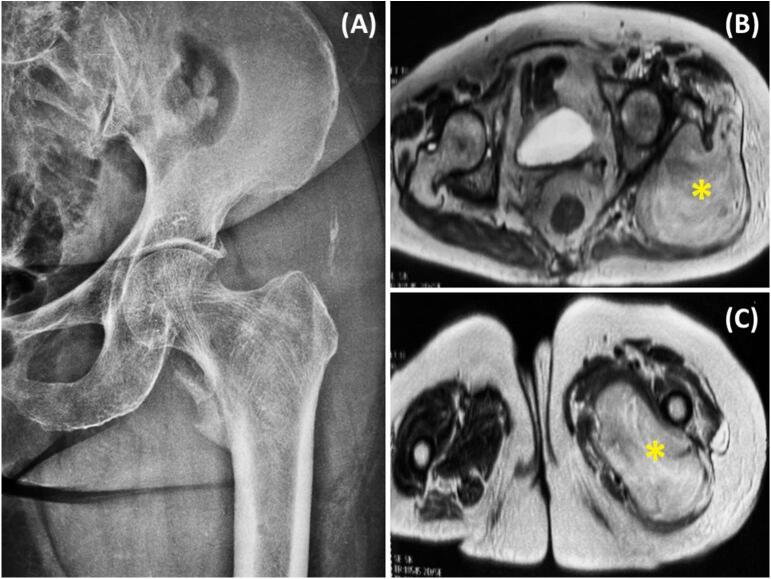
Radiographic and axial MRI findings. A: X-ray of the left hip showing well-defined osteolytic lesions involving the upper femur and proximal diaphysis, without peripheral condensation, and preservation of cortical bone. Associated soft tissue ossifications are noted near the greater and lesser trochanters. B: Axial T2-weighted MRI of the pelvis revealing a hyperintense soft tissue mass at the root of the left thigh infiltrating the gluteal and adductor muscles. C: Axial T1-weighted post-contrast MRI of the pelvis showing heterogeneous enhancement of the same mass. The lesion measures 148 × 118 mm in the axial plane and 228 mm in height (yellow asterisk).

**Fig. 2:**
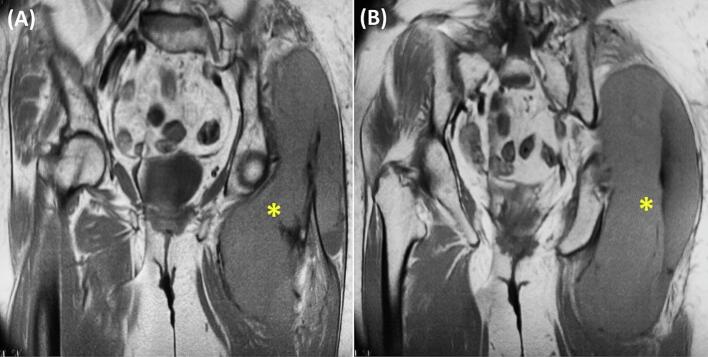
Coronal MRI findings. A: Coronal T1-weighted MRI of the pelvis showing a large soft tissue mass involving the gluteal and adductor muscles with mild hyperintensity. B: Coronal post-contrast T1-weighted MRI demonstrating heterogeneous enhancement and diffusion restriction of the mass, consistent with aggressive infiltration. Dimensions are 148 × 118 mm axially and 228 mm craniocaudally (yellow asterisk).

**Fig. 3:**
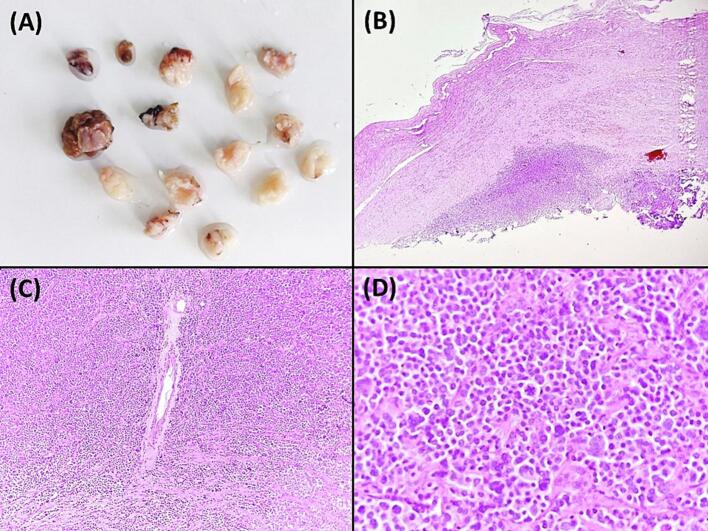
Gross and microscopic pathology. A: Gross appearance of the excised biopsy specimens: fifteen soft, whitish tissue fragments submitted for histological examination. B: Low-power view showing sheets of atypical plasma cells circumscribed by thick fibrous tissue (Hematoxylin and eosin, magnification ×40). C: Intermediate-power view displaying densely packed malignant plasma cells arranged in sheets (Hematoxylin and eosin, magnification ×100). D: High-power view highlighting cytological variability, including plasmablasts, bizarre multinucleated cells, flame cells, and Mott cells with perinuclear clearing (Hematoxylin and eosin, magnification ×400).

The X-ray of the left hip (Fig. 1A) revealed well-defined osteolytic lesions at the upper end of the femur and the proximal diaphysis, without peripheral condensation and preserving the cortical bone. Additionally, there were soft tissue ossifications near the greater and lesser trochanters. The MRI of the pelvis (Figs. 1B, C, 2A, B) revealed a large mass at the root of the left thigh, invading the gluteal and adductor muscles. This mass exhibited hyperintensity on T2-weighted images, mild hyperintensity on T1-weighted images, showed significant diffusion restriction, and enhanced heterogeneously after contrast injection. It measured 148 × 118 mm in the axial plane and extended 228 mm in height. The suggested diagnoses were amyloidoma or soft tissue sarcoma. A surgical biopsy of the soft tissue mass was conducted using a posterolateral approach to the hip, specifically targeting the tumor that was located through palpation. The specimens were forwarded to the pathology laboratory for analysis (Fig. 3A).

### 2.4 Pathology findings

Histological analysis of the biopsy specimens (Fig. 3B, C and D) revealed a malignant tumor proliferation circumscribed by a thick fibrous tissue. The lesion exhibited high cellularity, consisting of sheets of atypical plasma cells with eccentrically located nuclei and conspicuous perinuclear halos. Various cytological variants were observed, such as plasmablasts with vesicular nuclear chromatin and bizarre multinucleated cells. Additionally, cytoplasmic inclusions were noted, including flame cells with bright red cytoplasm and Mott cells with grapelike clusters of cytoplasmic droplets. The mitotic rate was elevated with multiple atypical mitotic figures. Congo red staining yielded negative results, confirming the absence of amyloid deposits. Immunohistochemical analysis confirmed positive immunostaining for CD138, CD38, MUM1, EMA and CD79a supporting the diagnosis of extramedullary myeloma.

### 2.5 Treatment and follow-up

The patient was referred for adjuvant radiotherapy; however, she succumbed to severe pneumonia shortly after completing the first session.

## 3 Discussion

MM, the most prevalent primary osseous malignancy in adults, typically affects individuals aged 50 to 70, with a higher incidence among men [1,2,5]. The reported incidence of extramedullary disease varies due to inconsistencies in its definition and diagnostic criteria [6]. Studies indicate its occurrence ranges from 1.7 % to 4.5 % in newly diagnosed MM patients and from 3.4 % to 14 % in those with relapsed disease [6,7]. In a large cohort study involving 3744 MM patients, approximately 14.5 % had extramedullary disease at diagnosis [8]. Extramedullary soft tissue masses have been documented in various anatomical sites, such as the gingiva, liver, pituitary gland, small intestine, breast, thyroid cartilage, heart, testis, stomach, and soft tissues of the neck [9]. Soft-tissue plasmacytomas are defined as plasma cell tumors arising in musculoskeletal structures such as muscle or subcutaneous fat, as well as in connective tissues, and are distinct from those involving visceral organs or lymph nodes. They represent an aggressive manifestation of MM, marked by the capacity of malignant clones or subclones to proliferate and persist independently of the bone marrow microenvironment. This aggressive behavior is associated with high-risk genetic alterations, increased proliferation, evasion of programmed cell death, and resistance to treatment [4,5]. Extramedullary involvement in MM has been associated with various genetic characteristics. These encompass 17p deletion [5], nuclear expression of p53 [4,5], an increased prevalence of t (4;14) [5], and p53 deletion [5]. Additionally, potential risk factors consist of diminished CD56 expression [5], heightened MAFB expression [5], and elevated MYC expression [5]. In patients with MM, soft-tissue plasmacytomas can develop through direct extension from skeletal tumors following cortical bone disruption, dissemination via the bloodstream leading to growth in organs or soft tissues without direct bone contact, or, rarely, due to invasive medical procedures [7,8]. Clinically, extramedullary lesions can manifest in three ways: as a tumor mass near bone extending into soft tissues, as a soft tissue or visceral tumor independent of bone connection, or as the diffuse infiltration of organs by plasma cells without clear focal lesions. However, the majority of studies tend to overlook the distinction between these three types of extramedullary lesions [10,11]. Imaging is pivotal in assessing patients with MM, aiding in discerning the effects of myeloma cells on the skeletal system and pinpointing extramedullary disease [12]. F18-FDG-PET/CT and MRI have recently emerged as indispensable modalities for the thorough evaluation and management of MM patients, providing additional advantages in clinical care [13]. F18-FDG PET/CT can be instrumental in highlighting regions of metabolic activity throughout the entire body, indicating sites of clonal plasma cell proliferation, whereas MRI is especially adept at visualizing the bone marrow [14]. MM featuring a sizable extramedullary plasma cell tumor frequently demonstrates limited responsiveness to chemotherapy and radiotherapy. For cases of progressively expanding masses, certain experts suggest surgical resection to diminish tumor burden, alleviate pain, and address potential secondary complications like vascular and nerve compression [15]. In our case, due to the patient's compromised overall health status, we decided against pursuing surgical intervention. The absence of large-scale prospective studies and expert consensus guidelines on the management of extramedullary disease poses significant challenges in determining optimal treatment strategies [8]. For non-transplant patients, regimens with daratumumab and VMP or RVD are recommended. For transplant-eligible patients, intensive therapies like VTD or VRD/PACE with stem cell transplantation are suggested [16]. For relapsed cases, lymphoma-like regimens such as PACE, DCEP, or Dexa-BEAM are used, though response lasts less than 4 months. Novel agents, including carfilzomib, selinexor, or isatuximab, as well as investigational therapies like CAR-T, BiTEs, and melflufen, are alternatives [16]. The involvement of soft tissues is linked to significantly reduce progression-free survival and overall survival, with a median survival of just 8.5 months [9,10]. Radiotherapy is a palliative option for localized extramedullary disease, particularly to alleviate pain or prevent complications like nerve compression. While it may reduce tumor burden, its efficacy is limited in disseminated disease. In this case, radiotherapy was initiated but interrupted due to the patient's terminal condition [4].

This case report presents several inherent limitations. First, the absence of comprehensive cytogenetic analysis, such as fluorescence in situ hybridization (FISH) for high-risk abnormalities (e.g., 17p deletion, 1q gain, or t(14;16)), limits the ability to establish correlations between genetic features and the aggressive clinical course observed. Additionally, as a single case report, the findings cannot be generalized to broader patient populations but serve to highlight the diagnostic complexity and clinical severity of rare extramedullary manifestations. The report is intended to enhance clinical awareness rather than draw definitive conclusions regarding prognosis or treatment strategies.

## 4 Conclusion

In conclusion, this case highlights the rarity and aggressive nature of extramedullary multiple myeloma, underscoring the considerable diagnostic and therapeutic challenges it presents. The unusual manifestation as a rapidly growing buttock mass, initially suspected to be an amyloidoma or sarcoma, makes this case particularly noteworthy. Early recognition, along with comprehensive imaging and histopathological examination, is crucial for accurately identifying unusual soft tissue involvement. Given the poor prognosis associated with extramedullary progression, further research is needed to refine treatment strategies and improve patient outcomes.

## Consent statement

Written informed consent was obtained from the patient for publication of this case report and accompanying images. A copy of the written consent is available for review by the Editor-in-Chief of this journal on request.

## Ethical approval

Ethical approval for this study was provided by the Ethical Committee of Mongi Slim University Hospital, Marsa, Tunisia.

## Guarantor

Dr. Faten LIMAIEM.

## Provenance and peer review

Not commissioned, externally peer-reviewed.

## Funding

This research did not receive any specific grant from funding agencies in the public, commercial, or not-for-profit sectors.

## Author contribution

Dr. Faten LIMAIEM: Prepared, organized, wrote, and edited all aspects of the manuscript.

Dr. Mohamed Amine GHARBI, and Pr. Ramzi BOUZIDI: Read, edited, and approved the final version of the manuscript. Contributed to data acquisition, analysis, and interpretation. Provided final approval of the manuscript before its submission.

## Declaration of competing interest

None declared.
